# Assessing Body Composition in Paralympians: Accuracy of Different Measurement Methods Compared with Dual-Energy X-Ray Absorptiometry

**DOI:** 10.3390/jfmk11010001

**Published:** 2025-12-19

**Authors:** Raffaella Spada, Valentina Cavedon, Lucrezia Macori, Amedeo Toraldo, Loredana Torrisi, Alessio Franco, Maria Rosaria Squeo, Marco Bernardi

**Affiliations:** 1 Institute of Sports Medicine and Science, National Italian Olympic Committee, Largo Piero Gabrielli, 1, 00197 Rome, Italy; ext_raffaella.spada@coni.it (R.S.); lucreziamacori@gmail.com (L.M.); dottoressatorrisi@gmail.com (L.T.); alessio.franco@gmail.com (A.F.); mariarosaria.squeo@coni.it (M.R.S.); 2 Department of Neurosciences, Biomedicine and Movement Sciences, University of Verona, 37131 Verona, Italy; 3 Department of Science and Technology for Humans and the Environment, Università Campus Bio-Medico di Roma, 00128 Rome, Italy; amedeo0201@gmail.com; 4 Department of Physiology and Pharmacology “V. Erspamer”, Sapienza University of Rome, 00185 Rome, Italy; marco.bernardi@uniroma1.it

**Keywords:** paralympic sports, adapted sports, bioelectrical impedance analysis, air displacement plethysmography, skinfold thickness, skinfold predictive equations, spinal cord injury, limb amputations, physical impairment, DXA

## Abstract

**Background**: Paralympic athletes represent a highly heterogeneous athletic population, which poses unique challenges for body composition assessment. This study evaluated the accuracy of Bioelectrical Impedance Analysis (BIA), Air Displacement Plethysmography (ADP), and a set of skinfold equations in estimating relative fat mass (%FM) in Paralympians, using Dual-Energy X-Ray Absorptiometry (DXA) as reference method. **Methods**: Sixty-six male and sixty-seven female Paralympians underwent body composition assessments on the same day. The %FM estimated using BIA, ADP, and six existing skinfold equations was compared with %FM measured by DXA (%FM_DXA). Accuracy and agreement between the methods were evaluated using two-tailed paired-sample *t*-tests, concordance correlation coefficients, reduced major axis regression, and Bland–Altman analysis. Linear regression analyses with the %FM_DXA as dependent variable and anthropometric measurements as independent variable were also carried out. **Results**: BIA, ADP, and skinfold equations exhibited poor agreement with DXA and significantly underestimated %FM_DXA, with systematic biases ranging from −1.8% to −10.7% in both men and women. In both groups, skinfold sums showed strong correlations with %FM_DXA (r > 0.7), with the nine-skinfold model providing the best prediction (adjusted R^2^ approximately 0.8). **Conclusions**: The results of this study indicate a lack of transferability of available methods for assessing body composition (skinfold equations, BIA, and ADP) in estimating %FM_DXA in both male and female Paralympians, as these methods proved inaccurate. Future research is needed to further investigate the accuracy of methods for assessing body composition in this population, taking into account the specific impairment and health condition of the athletes.

## 1. Introduction

In recent years, the participation of athletes with an impairment in high-profile international events, such as the Paralympic Games [1,2,3], has significantly increased, alongside the growing professionalism of these athletes [4]. Similarly to Olympic sports, this has led to an increasing attention on the periodic health and fitness evaluation of potential Paralympic candidates (hereinafter referred to as Paralympians) [5,6,7,8,9,10]. Paralympic athletes (PA) often experience a range of distinct impairments and health conditions that contribute to the complexity of the sporting environment [10]. This calls for more detailed information on each athlete and may require more complex strategies to address, prevent, and mitigate health concerns [11,12].

The assessment of body composition has become routine among PA, with applications in general health screenings to evaluate nutritional status and in the monitoring of the impact of dietary and training interventions [13]. PA represent an athletic population with inherent variability, including individuals with different types and degrees of physical, visual, and intellectual impairments [14]. While the principles for assessing body composition in athletes with intellectual and vision impairments largely align with those for able-bodied athletes, PA with an impairment such as reduced muscle power (e.g., those with a spinal cord injury), limb deficiency (e.g., those with amputations), or restricted passive range of movement (e.g., those with arthrogryposis or scoliosis) pose unique challenges in body composition assessment [15]. In particular, it has been reported that PA with physical impairments exhibit a specific body composition profile that differs from that of their able-bodied counterparts, with distinct patterns of tissue distribution across body regions that can vary based on several factors, including the type of the impairment, the sport practiced, the amount of training, and the duration of injury [16,17,18,19,20,21,22].

Overall, and of special interest from a health-related perspective, PA, especially those with physical impairments, experience changes in body composition due to their conditions, including increased whole-body adiposity, muscle atrophy, reduced lean mass and bone mineral density, as well as an increase in fat mass [18,23,24,25]. Accordingly, accurately assessing and monitoring body composition over time in PA is particularly important, as such alterations, including the accumulation of fat mass in specific body regions, are closely associated with other conditions, such as osteoporosis, obesity, cardiovascular disease, hypertension, type II diabetes mellitus, certain forms of cancer, and an increased risk of mortality [26,27].

The assessment of body composition is also important from a performance perspective. Body composition characterizes not only PA competing in endurance sports, but also those involved in power, mixed metabolism, and skill sports [28]. In particular, among endurance PA, evidence indicates that the most successful athletes (i.e., Paralympic medalists) exhibit lower fat mass values than their peers, suggesting that fat mass percentage is associated with performance [28].

Techniques for assessing body composition vary in complexity and setting, ranging from laboratory-based to field-based methods, each presenting advantages and limitations, along with some degree of measurement error [15,29]. Various techniques for assessing body composition include manual anthropometry (such as body circumferences and skinfold thickness), Bioelectrical Impedance Analysis (BIA), Air Displacement Plethysmography (ADP), Hydrostatic Weighing, Dual-Energy X-ray Absorptiometry (DXA), and imaging methods like Computed Tomography and Magnetic Resonance Imaging [30]. The choice of method typically depends on the intended goal of the measurements and the technology available.

Today, DXA has been recognized as an accurate method to assess body composition in PA [29,31,32]. However, DXA is not always accessible in many sports contexts due to logistical challenges and costs. As a result, clinicians, nutritionists, and physical conditioners often turn to more accessible methods, such as BIA or anthropometric predictive equations, as alternatives for estimating body composition in PA. However, the accuracy of these methodologies relies on predictive equations based on the assumption that body tissues are distributed in a similar manner across all individuals within a given population and are specific to the population being evaluated [33]. The current body composition methodologies based on predictive equations have been mostly validated for able-bodied individuals, with few predictive models specifically developed in large cohorts of PA.

Despite the importance of precisely assessing body composition in this special athletic population, the accuracy of the currently available methods for estimating body composition in PA relative to DXA has been poorly investigated to date [19,32,34,35,36]. These studies assessed the accuracy of anthropometry [19,32,34,35,36], BIA [32,34], and ADP [32] in relatively small and often heterogeneous samples of PA. Taken together, these studies indicate a lack of transferability of the currently used methods for assessing body composition in PA when compared to DXA. They also highlight the need for further research that involves larger samples of PA, in order to better account for the impact of different types of impairments.

The present study was designed to address gaps in the literature by assessing body composition using various currently available methods in a large cohort of male and female Paralympians. These athletes were examined as part of the preparticipation medical program implemented at the Institute of Sport Medicine and Science of the Italian Olympic Committee [9,37]. The first aim of this study was to evaluate the accuracy of selected skinfold equations, BIA, and ADP in estimating body composition, using DXA as the reference method. A second aim of this study was to examine the association between anthropometric measures, BIA and ADP estimates, and relative fat mass as determined by DXA.

## 2. Materials and Methods

### 2.1. Participants

The Institute of Sport Medicine and Science conducts medical and performance evaluations of elite Italian athletes before their participation in the Paralympic Games [9,37]. This pre-participation medical assessment includes clinical, laboratory and instrumental tests that encompass the assessment of body composition, nutritional status and cardiovascular function. In this context, a total of 86 male (mean age 33.0 ± 10.0 years) and 75 female (mean age 32.8 ± 9.5 years) Paralympians were included in the study.

Recruited PA participated in 17 sport disciplines, that is Para Equestrian (*n* = 5), Para Shooting (*n* = 6), Para Archery (*n* = 14), Para Athletics (e.g., wheelchair racing, high jump, shot put; *n* = 17), Para Judo (*n* = 4), Para Swimming (*n* = 32), Para Powerlifting (*n* = 3), Para Badminton (*n* = 1), Sitting Volleyball (*n* = 14), Wheelchair Fencing (*n* = 12), Para Taekwondo (*n* = 1), Wheelchair Tennis (*n* = 2), Para Table Tennis (*n* = 7), Para Rowing (*n* = 9), Para Canoe (*n* = 6), Cycling (*n* = 21), and Para Triathlon (*n* = 7).

The study was conducted in accordance with the Declaration of Helsinki, and the protocol was approved by the Institutional Review Board of the local Institute of Sport Medicine and Science. The study design of the present investigation was evaluated and approved by the Ethical Committee (CET—Comitato Etico Territoriale Lazio Area 1, date of approval 6 March 2024 IRB number 0208/2024). All athletes involved in this study were thoroughly briefed on the purpose and procedures of the evaluation and provided their written informed consent, in compliance with Italian regulations and the policies of the Institute.

### 2.2. Procedures

All Paralympians underwent a body composition assessment between one and eight months before the Paris 2024 Paralympic Games. Body composition was assessed using a whole-body DXA scan, BIA, ADP, and anthropometric measurements. All measurements were conducted on the same day in the morning, performed consecutively, following a 3–4 h fast. Athletes were instructed to void their bladder prior to testing and to refrain from strenuous exercise during the day preceding the assessments to standardize hydration status. During all assessments, athletes wore minimal clothing (i.e., underwear). They were advised to refrain from vigorous physical activity on the day before the testing session and to avoid any exercise on the morning of the evaluations.

Prior to the body composition assessment, athletes completed a face-to-face questionnaire to collect general information, including age, sport practiced, and health condition.

#### 2.2.1. Anthropometric Assessment

The anthropometric evaluation involved the measurement of body mass, stature, skinfold thicknesses, and body circumferences.

Body mass was measured to the nearest 0.1 kg with athletes wearing only underwear, using a certified mechanical scale with stadiometer (SECA 711, GmbH, Hamburg, Germany). Athletes with upper limb amputation(s) were asked to remove their prosthesis before having their body mass measured. For athletes with lower limb amputation(s), body mass was measured with the prosthesis on, and the weight of the prosthesis was then subtracted from the total measurement to obtain the actual body mass. Similarly, for athletes unable to stand, body mass was assessed while seated on their wheelchair, with the wheelchair weight subtracted from the measurement to obtain the athlete’s actual body mass.

Standing height was measured to the nearest 0.1 cm using a mechanical scale with a stadiometer (SECA 711, GmbH, Hamburg, Germany) according to conventional criteria and measuring procedures [38]. Athletes with lower limb amputation(s) were measured while wearing their prosthesis, while for athletes who were wheelchair users and unable to stand, body stature was self-reported. Body mass index for all athletes was calculated as body mass (kg) divided by height squared (m^2^).

Body circumferences were measured using a non-elastic steel millimeter measuring tape (Anthroflex NA305, 6 mm × 2 m, Nutriactiva, Minneapolis, MN, USA). Based on the impairment, where the anatomical measurement sites were present, the following body circumferences were measured according to standard procedures [38]: waist, abdomen, and hips.

Skinfold thicknesses were measured to the nearest 0.2 mm by the same trained investigator with a mechanical skinfold caliper (GIMA, Milan, Italy). Annual calibration checks have been performed. Wherever compatible with the athlete’s impairment, skinfold thicknesses were measured at the biceps, triceps, subscapular, chest, axilla, suprailiac (anterior and medial), abdominal, anterior thigh, and calf according to conventional criteria and measurement procedures [38]. For athletes with an impairment affecting one side of the body, measurements were taken on the non-impaired side.

Body density or the %FM were calculated using four commonly used skinfold equations developed in able-bodied populations: Durnin and Womersley, 1974 (%FM_DW); Evans et al., 2005 (%FM_EV3 and %FM_EV7); Pollock et al., 1976 (%FM_POL); Thorland et al., 1984 (%FM_THO3 and %FM_THO) [39,40,41,42] (Table 1). Body density values were converted to %FM according to Siri [43].

In our laboratory, the technical error of measurement (%TEM) for the sum of nine skinfolds was 1.49%, and the intraclass correlation coefficient (ICC, two-way random effects, absolute agreement) was 0.99.

#### 2.2.2. Bioelectrical Impedance Analysis (BIA) Assessment

Bioelectrical impedance analyses were performed with impedance analyzer (BIA 101 BIVA PRO, AKERN srl, Pisa, Italy) at a single frequency of 50 kHz, with tetrapolar technique. The device was calibrated every morning using the standard control circuit supplied by the manufacturer. The accuracy of the device was 0.1% for resistance and 0.1% for reactance. Athletes were instructed to remove their socks and jewelry, then lie in a supine position on a non-conductive surface (e.g., a crib mattress or examination table) for 10 min to ensure procedural standardization. The arms were extended and positioned slightly apart from the body, ensuring that the ankles and thighs remained separated. Adhesive electrodes were placed on the athletes’ dorsal wrist and ankle after the skin was cleaned with alcohol. The wrist measurement landmark was located at the distal end of the third metacarpal and between the styloid processes of the radius and ulna. The ankle landmark was positioned at the end of the second metatarsal and between the medial and lateral malleoli. For athletes with unilateral upper (or lower) limb amputation on the right side, the left hand (or foot) was used. Athletes with bilateral lower limb amputation did not undergo this technique. It should be noted that the proprietary AKERN algorithms assume all body segments are present; therefore, in athletes with limb amputations, estimates of whole-body composition may be biased.

Relative body fat estimates were determined using the proprietary algorithms included in the AKERN software (Bodygram Pro, version 4.5.2). No additional analytical corrections were applied beyond those automatically implemented by the software.

For BIA, short-term test–retest reliability in our laboratory showed excellent precision for Resistance: TEM values ranged from 0.13 to 0.19 ohm (%TEM 0.04% to 0.18%), and ICC values ranged from 0.998 to 0.999.

#### 2.2.3. Air Displacement Plethysmography (ADP) Assessment

When possible (36 Female and 27 Male athletes), according to the impairment of the athlete, the relative fat mass (%FM_ADP) was assessed using a commercially available device (COSMED USA Inc., Concord, CA, USA) according to the procedures recommended by the manufacturer [44]. A two-point calibration was performed with an empty chamber before each athlete’s assessment. Athletes were instructed to wear tight-fitting swimwear and a snug nylon cap to cover their hair, and to remove any prostheses and metal objects, including jewelry. Once in the chamber, athletes were asked to remain still for the duration of the assessment. Two measurements of body volume were obtained during the ADP procedure. If the difference between the readings exceeded 150 mL, a third measurement was taken. Body volume was determined on the basis of the air displaced when the athlete was positioned in the chamber. Thoracic Gas Volume was predicted by the software using standard equations based on age, gender, and height. The COSMED software version 5.2.1 was used, and body density was calculated applying standard density constants (fat-free mass = 1.100 g/cm^3^, fat mass = 0.9007 g/cm^3^). Relative fat mass (%FM_ADP) was then derived from body density using the Siri equation, according to gender and age. No additional corrections beyond those automatically applied by the COSMED software were made. For athletes who were unable to remain seated without support (e.g., those with a spinal cord injury at the cervical or high thoracic level), this technique was not employed.

#### 2.2.4. Dual-Energy X-Ray Absorptiometry (DXA) Assessment

DXA-measured body composition was assessed through a whole-body scan on a QDR Explorer fan beam densitometer (Hologic, MA, USA). In the laboratory, daily quality control of the DXA scanner was performed prior to scanning with an encapsulated spine phantom (Hologic Inc., Bedford, MA, USA) to monitor potential baseline drift. Throughout the testing period, the device exhibited negligible baseline drift. Whole-body DXA scans were conducted on participants according to the guidelines outlined in “The Best Practice Protocol for the Assessment of Whole-Body Body Composition by DXA” [45]. Before the scan, athletes were instructed to remove any metal objects, jewelry, or reflective materials, including prosthetics. Positioning aids were utilized to support the residual lower limb, and special strapping was applied around the residual ankle to ensure stability during the scans. The “standard” scan mode was selected prior to each scan. All DXA scans were analyzed by the same trained investigator to maintain consistency. The analysis of DXA scans was carried out with the Hologic Discovery software (version 12.6.1) for Windows XP, in line with the manufacturer’s guidelines. For the purposes of this study, the whole-body relative fat mass (%FM_DXA) was used for analysis.

#### 2.2.5. Statistical Analysis

The normality of data was assessed using the Shapiro–Wilk test and descriptive statistics (mean and standard deviation) were computed for all variables, according to the requirements of each test. For paired *t*-tests, normality was specifically evaluated on the paired differences between methods, as required by the test assumptions.

Mean bias (i.e., the average of the differences between the %FM obtained by each skinfold equation and the %FM_DXA) and its 95% confidence intervals (CIs) were computed to get a measure of systematic measurement errors. Non-parametric variables were log-transformed and the %FM obtained by each skinfold equation was compared with the %FM_DXA using the two-tailed paired-sample *t*-test. Effect sizes for paired *t*-tests were calculated using Cohen’s d and interpreted as small (0.2), medium (0.5), and large (0.8) according to Cohen’s guidelines [46].

The Lin’s concordance correlation coefficient (ρc) was used to quantify the agreement between the %FM_DXA and each skinfold equation [47]; agreement was considered poor (ρc < 0.90), moderate (ρc between 0.90 and 0.95), substantial (ρc between 0.95 and 0.99), excellent (ρc > 0.99) and perfect (ρc = 1) [47]. The Reduced Major Axis (RMA) regression [48] was used to assess the relationship between the %FM_DXA (i.e., the dependent variable) and the %FM predicted by each skinfold equation. In case of perfect agreement, the intercept and the slope of the RMA line are 0 and 1, respectively. Agreement between each skinfold equation and DXA was tested using Bland–Altman analysis (limits of agreement and range) [49].

Missing data were handled using pairwise deletion. That is, each comparison between two methods included only the participants with available measurements for both methods, allowing maximal use of the available data without excluding cases unnecessarily.

The strength of the relationship between %FM_DXA and each individual anthropometric measurement was estimated using Pearson’s correlation coefficient (r_P_) for normally distributed variables and Spearman’s correlation coefficient (r_s_) for non-normally distributed variables.

Linear regression analyses with the %FM_DXA as dependent variable and each individual anthropometric measurement as independent variable were also carried out. The adjusted coefficient of determination (Adj R^2^) and the standard error of estimate (SEE) were used to assess the goodness-of-fit of each predictive model. Each regression model was validated using a repeated five-fold cross-validation (1000 repetitions), estimating for each test fold the root mean squared prediction error (RMSPE) and the coefficient of determination (R^2^ CV).

Statistical analyses were performed using SPSS v. 16.0 (IBM Corp., Armonk, NY, USA) and R-4.5.2 (Foundation for Statistical Computing, Vienna, Austria). The statistical significance was set at *p* ≤ 0.05.

## 3. Results

**Figure 1:**
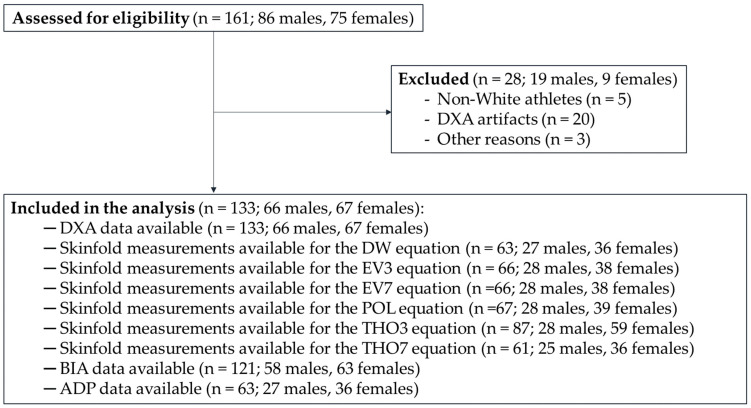
CONSORT flow diagram of the study. Legend: DXA, Dual-Energy X-ray Absorptiometry; DW, Durnin and Womersley; EV3, three-site equation by Evans and colleagues; EV7, seven-site equation by Evans and colleagues; POL, Pollock and colleagues; THO3, three-site equation by Thorland and colleagues; THO7, seven-site equation by Thorland and colleagues; BIA, Bioelectrical Impedance Analysis; ADP, Air Displacement Plethysmography.

The majority of athletes were of Caucasian descent (*n* = 156; 97%), and the sample did not sufficiently represent other races, such as Asian, Hispanic, or Black. Given the impact of race on body composition [50], two athletes of Asian descent and three athletes of Black descent were excluded from the analysis. The rationale for excluding them, rather than retaining all participants and including race as a covariate or presenting sensitivity analyses, is that the small subgroup sizes of non-White athletes preclude such analyses. Additionally, twenty athletes were excluded due to the presence of DXA artifacts (e.g., spasms during data acquisition, inability to maintain the required position, or the presence of metal implants). Finally, three athletes were excluded for other reasons (e.g., incomplete dataset). Accordingly, a total of 133 Paralympians (66 males and 67 females) were included in the analysis. Figure 1 shows the CONSORT flow diagram of the study.

**Figure 2:**
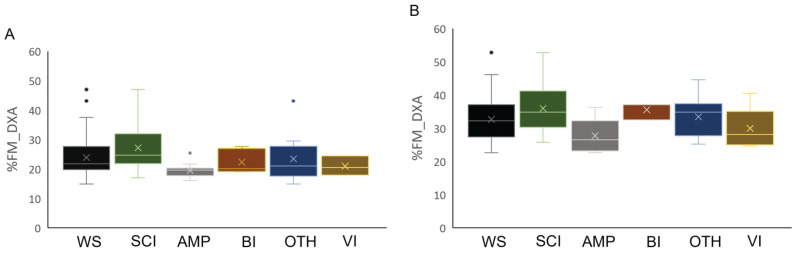
Box plot of relative fat mass estimated by DXA in male (Panel (**A**)) and female (Panel (**B**)) athletes in the total sample stratified by health condition. Legend: %FM_DXA, whole-body relative fat mass; WS, whole sample; SCI, Spinal Cord Injuries and Related Disorders Group; AMP, Amputation Group; BI, Brain Injury Group; OTH, Other Health Conditions Group; VI, Vision and Intellectual Health Conditions Group.

**Figure 3:**
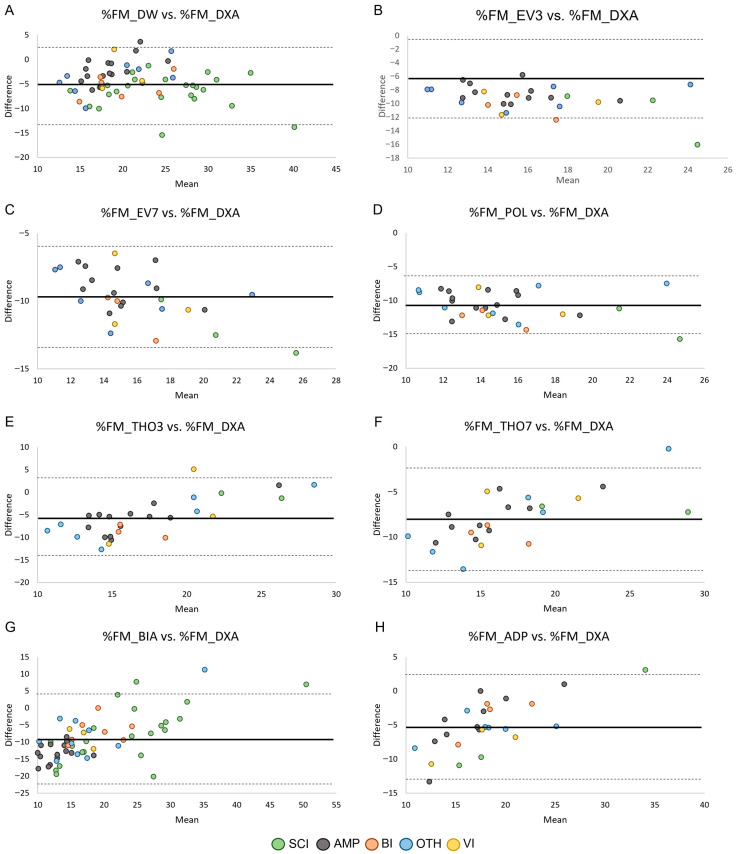
Bland–Altman plots showing the agreement by health condition between the relative fat mass assessed through skinfold equations (Panels (**A**–**F**)), BIA (Panel (**G**)), and ADP (Panel (**H**)) and the relative fat mass assessed through DXA in males. Data points are color-coded according to health condition group, while panels reflect measurement methods. Sample sizes for each comparison are reported in Table 3. Legend: SCI, Spinal Cord Injuries and Related Disorders Group; AMP, Amputation Group; BI, Brain Injury Group; OTH, Other Health Conditions Group; VI, Vision and Intellectual Health Conditions Group. The solid lines indicate bias ± 2 standard deviations.

**Figure 4:**
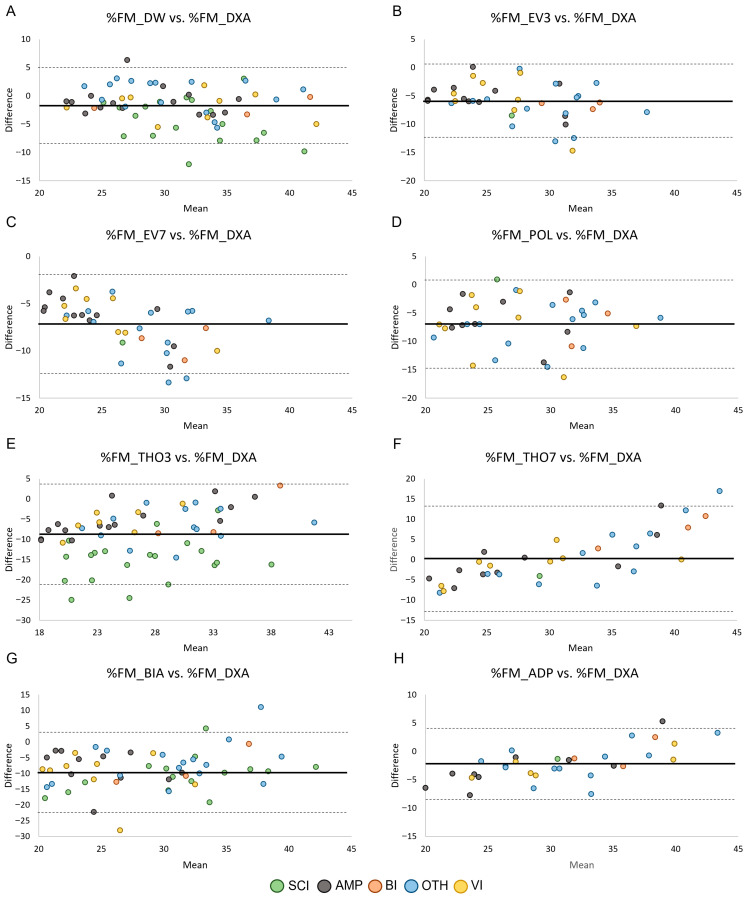
Bland–Altman plots showing the agreement by health condition between the relative fat mass assessed through skinfold equations (Panels (**A**–**F**)), BIA (Panel (**G**)), and ADP (Panel (**H**)) and the relative fat mass assessed through DXA in female PA. Data points are color-coded according to health condition group, while panels reflect measurement methods. Legend: SCI, Spinal Cord Injuries and Related Disorders Group; AMP, Amputation Group; BI, Brain Injury Group; OTH, Other Health Conditions Group; VI, Vision and Intellectual Health Conditions Group. The solid lines indicate bias ± 2 standard deviations.

In the descriptive analyses reported in Figure 2, Figure 3 and Figure 4, athletes were divided into five distinct health condition groups: the Spinal Cord Injuries and Related Disorders Group (SCI; *n* = 52; male PA, *n* = 29 and female PA, *n* = 23), which included athletes with spinal cord injury (*n* = 47), or spina bifida (*n* = 5); the Amputation Group (AMP; *n* = 31; males, *n* = 16 and females, *n* = 15), which included athletes with a total or partial absence of bones or joints in the upper limb (*n* = 7), lower limb(s) (*n* = 22), or both (*n* = 2), resulting from trauma, illness, or congenital limb deficiency; the Brain Injury Group (BI; *n* = 10; male PA, *n* = 7 and female PA, *n* = 3), which included athletes with acquired brain injuries or other neurological conditions related to brain damage, such as cerebral palsy; the Other Health Conditions Group (OTH; *n* = 28; male PA, *n* = 11 and female PA, *n* = 17), which included athletes with health conditions different from those included in the other groups, such as multiple sclerosis, femoral dysplasia, agenesis, and arthrogryposis; the Vision and Intellectual Health Conditions Group (VI; *n* = 12; male PA, *n* = 3 and female PA, *n* = 9), which included athletes with vision impairment and intellectual impairment.

Descriptive statistics (mean ± standard deviation) for age, body mass, stature, and BMI of male and female participants across groups are presented in Table 2. Figure 2 illustrates the %FM_DXA of the whole sample of male and female athletes (panels A and B, respectively), as well as by health condition groups.

A summary of the accuracy and agreement in the estimation of %FM by each skinfold equation, BIA, and ADP compared with %FM_DXA is presented in Table 3. The *t*-test showed that only %FM_THO7 in the female group was not significantly different from the average %FM_DXA, whereas all the other skinfold equations and field methods (i.e., BIA and ADP) significantly underestimated %FM_DXA, with systematic bias ranging from −1.78% (%FM_DW in the female group) to −10.69% (%FM_POL in the male group).

**Table 3:** Agreement between relative fat mass obtained using skinfold equations, BIA, and ADP, and that measured by DXA (reference method) in Paralympians.

	Descriptive Statistics		Paired *t*-Test				CCC	RMA Regression		Bland–Altman Analysis		
Method	Mean	SD	Bias (95% CI)	t	*p*	ES	ρc (95% CI)	Slope	Int	U LoA (95% CI)	L LoA (95% CI)	Range
Males												
%FM_DW (*n* = 59)	19.17	5.80	−4.55 (−5.48; −3.62)	9.59	<0.001	−1.28	0.64 (0.51; 0.74)	0.92	−2.71	2.43 (0.8; 3.86)	−11.53 (−13.33; −9.54)	13.96
%FM_EV3 (*n* = 28)	11.49	3.26	−9.23 (−10.01; −8.45)	22.93	<0.001	−4.55	0.20 (0.10; 0.29)	0.81	−5.36	−5.25 (−6.97; −5.12)	−13.21 (−15.08; −11.29)	7.95
%FM_EV7 (*n* = 28)	11.04	2.99	−9.68 (−10.44; −8.92)	26.47	<0.001	−5.07	0.17 (0.09; 0.26)	0.74	−4.38	−5.94 (−6.97; −5.12)	−13.42 (−14.42; −12.14)	7.47
%FM_POL (*n* = 28)	9.91	3.42	−10.69 (−11.03; −9.35)	21.90	<0.001	−4.72	0.16 (0.08; 0.24)	0.85	−7.64	−6.46 (−7.57; −5.58)	−14.91 (−16.11; −13.47)	8.45
%FM_THO3 (*n* = 28)	14.53	6.32	−5.66 (−7.22; −4.10)	6.70	<0.001	−1.30	0.38 (0.20; 0.53)	1.92	−24.25	2.90 (−0.43; 5.74)	−14.22 (−16.01; −12.02)	17.12
%FM_THO7 (*n* = 25)	12.64	5.83	−8.02 (−9.20; −6.84)	9.18	<0.001	−2.80	0.35 (0.20; 0.49)	1.48	−17.91	−2.40 (−4.91; −0.13)	−13.64 (−15.01; −11.68)	11.25
%FM_BIA (*n* = 58)	13.97	10.18	−9.16 (−10.96; −7.36)	10.38	<0.001	−1.36	0.42 (0.30; 0.53)	1.69	−25.21	4.01 (−0.36; 8.04)	−22.33 (−24.56; −19.76)	26.34
%FM_ADP (*n* = 27)	15.10	6.63	−5.33 (−6.53; −4.13)	5.72	<0.001	−1.37	0.50 (0.31; 0.64)	1.71	−19.74	2.31 (−0.27; 4.44)	−12.97 (−15.02; −10.47)	15.27
Females												
%FM_DW (*n* = 61)	29.99	5.09	−1.78 (−2.26; −1.30)	4.03	<0.001	−0.51	0.76 (0.64; 0.85)	0.86	2.54	4.99 (3.62; 6.29)	−8.56 (−10.33; −6.78)	13.55
%FM_EV3 (*n* = 38)	24.50	4.20	−5.92 (−6.91; −4.93)	10.96	<0.001	−1.78	0.42 (0.26; 0.56)	0.80	0.27	0.60 (−1.12; 2.07)	−12.44 (−14.58; −9.97)	13.04
%FM_EV7 (*n* = 38)	23.26	3.82	−7.15 (−8.03; −6.27)	16.45	<0.001	−2.67	0.37 (0.24; 0.49)	0.72	1.24	−1.90 (−3.06; −0.85)	−12.41 (−13.84; −10.60)	10.51
%FM_POL (*n* = 39)	23.71	5.52	−6.96 (−8.28; −5.64)	10.91	<0.001	−1.75	0.40 (0.24; 0.54)	1.01	−7.33	0.85 (−0.82; 2.22)	−14.77 (−17.01; −12.28)	15.62
%FM_THO3 (*n* = 59)	22.43	7.49	−9.00 (−10.66; −7.34)	10.84	<0.001	−1.41	0.28 (0.15; 0.40)	1.31	−18.65	3.50 (0.98; 5.52)	−21.51 (−24.66; −18.24)	25.01
%FM_THO7 (*n* = 36)	30.34	10.34	0.13 (−2.27; −2.01)	−0.12	0.909	−0.02	0.67 (0.55; 0.76)	2.02	−30.67	13.08 (8.45; 16.98)	−12.82 (−14.93; −10.06)	25.90
%FM_BIA (*n* = 63)	22.90	8.44	−9.56 (−11.47; −7.65)	11.66	<0.001	−1.47	0.35 (0.22; 0.47)	1.27	−18.23	3.20 (−0.20; 6.35)	−22.31 (−25.33; −19.02)	25.51
%FM_ADP (*n* = 36)	29.43	8.03	−2.33 (−3.21; −1.45)	4.38	<0.001	−0.73	0.85 (0.76; 0.90)	1.39	−14.57	4.00 (2.07; 5.54)	−8.48 (−9.98; −6.83)	12.48

Legend: SD, standard deviation: CI, confidence interval; *p*, *p*-value; ES, effect size; CCC, concordance correlation coefficient; RMA, reduced major axis; ρc, Lin’s concordance correlation coefficient; Int, intercept; U, upper; L, lower; LoA, limits of agreement.

The ρc indicated poor agreement between %FM obtained with each of the considered skinfold equations and field methods and %FM_DXA (ρc < 0.90 for all, Table 3). The slope and the intercept of the RMA were, respectively, near to 1 and to 0 for the %FM_DXA and the %FM_EV3 in female PA indicating good concordance between these measurements. All other measurement showed poor agreement (Table 3).

The agreement between %FM obtained by ADP and field methods (i.e., skinfold equations, BIA) and %FM_DXA in the male and female groups is shown in Figure 3 and Figure 4, respectively. Across methods, all but one data point per comparison fell within the 95% limits of agreement, corresponding to proportions between 96.6% and 100% across panels (exact sample sizes are reported in Table 3). However, despite the high proportion of observations within the limits, the width of the limits of agreement was large (range: 7.47–26.34 in male PA and 10.51–25.90 in female PA), indicating poor agreement between methods.

**Figure 5:**
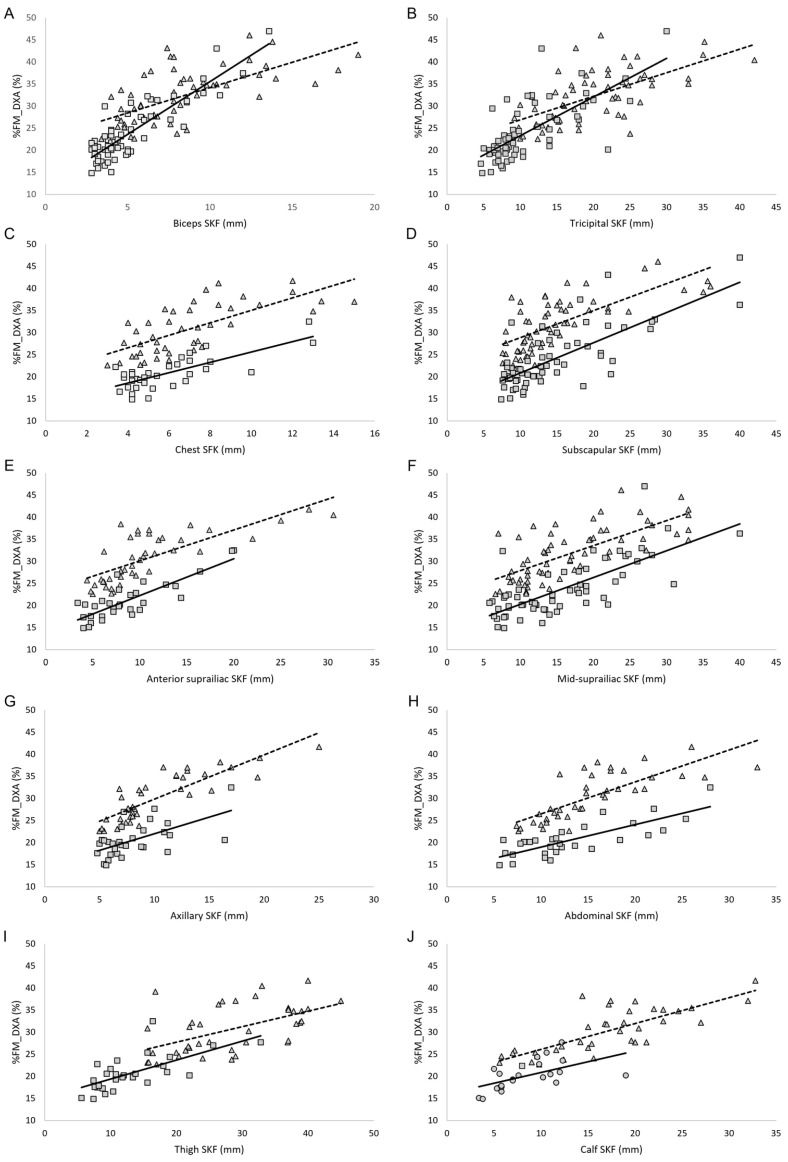
(**A**–**J**) Correlation between skinfold thicknesses and relative fat mass assessed by DXA in male PA (squares) and female PA (triangles). Legend: %FM_DXA, relative fat mass assessed though DXA; SKF, skinfold; the solid line represents the regression line in the male group; the dashed line represents the regression line in the female group.

For each regression model, only athletes with available measurements for the corresponding predictor were included, meaning that the sample size could differ between models. Detailed numbers of participants for each predictor are provided in Table 4. For these participants, we computed the Pearson correlation coefficient (r), the coefficient of determination (R^2^), the adjusted R^2^, and the standard error of the estimate (SEE) to assess the strength and accuracy of each predictor. As shown in Table 4 and Figure 5, the correlation analysis revealed a positive and statistically significant association between each skinfold thickness and %FM_DXA in both groups. In male PA, the highest correlation between each skinfold thickness and %FM_DXA was observed for the biceps skinfold, and the lowest for the calf skinfold. In female PA, the highest correlation was found for the axillary skinfold, whereas the lowest was for the thigh skinfold. In both sexes, waist circumference showed the strongest correlation with %FM_DXA (Table 4). Moreover, in both males and females, the sums of four, seven, and nine skinfolds showed positive and statistically significant correlations with %FM_DXA, with correlation coefficients greater than 0.7 (Table 4). In both groups, the sum of nine skinfolds provided the highest predictive power for %FM_DXA (adjusted R^2^ = 0.76 in male PA and 0.79 in female PA), along with the lowest standard error of estimate (1.54 in male PA and 2.37 in female PA). Overall, the sum of skinfolds (sums of three, seven, and nine skinfolds) and the biceps skinfold showed the highest predictive performance, with R^2^ CV values ranging from 0.70 to 0.84 in males and 0.58 to 0.84 in females, and RMSPE values generally below 4.0%FM. Single skinfolds such as the thigh and calf skinfolds had lower predictive performance, particularly in females (R^2^ CV 0.35–0.62, RMSPE 3–5%FM). Waist, abdominal, and hip circumferences demonstrated moderate predictive ability (R^2^ CV 0.25–0.60).

## 4. Discussion

Accurate assessment of body composition in athletes with impairments is crucial in Paralympic sports, given its relationship with both health and athletic performance. Unfortunately, however limited evidence exists in the literature regarding the ability of field measurements to accurately assess body composition in PA, particularly in female PA [19].

The primary aim of this study was to evaluate the ability of ADP and field methods (i.e., existing skinfold equations validated in able-bodied populations, and BIA) to estimate %FM in male and female Paralympians, using DXA as the reference standard. The results showed that nearly all skinfold equations developed in able-bodied populations, as well as BIA and ADP, failed to accurately estimate %FM_DXA in both male and female Paralympians. In females, %FM_THO7 was the only skinfold equation that predicted %FM values close to %FM_DXA, with a mean bias of 0.13%; this difference was not statistically significant (Table 3). However, the Bland–Altman plot (Figure 4) showed that although nearly all data points fell within the 95% limits of agreement, the limits were very wide, indicating poor prediction accuracy. For all the other skinfold equations, the two-tailed paired-sample *t*-test revealed a significant systematic bias, with %FM_DXA being underestimated in both male and female Paralympians (Table 3). In male PA, a large (≥ 4%) systematic bias was observed in all the evaluated skinfold equations, whereas in female PA it was found in 4 out of 6 skinfold equations. These findings are consistent with previous studies [32,34,36] indicating that anthropometry for assessing %FM in PA systematically underestimates %FM_DXA. This suggests that skinfold equations developed in able-bodied populations are not adequate for accurately predicting %FM_DXA in male and female Paralympians. Consequently, our results highlight the need for specific skinfold equations tailored to this athletic population.

Our results showed that BIA consistently underestimated %FM compared with DXA, with large variations in BIA estimates and poor agreement in both male and female PA (Table 3 and Figure 3 and Figure 4). Our findings align with previous research [32,34] and suggest that BIA prediction equations are not suitable for this athletic population. The variability observed may be attributable to pronounced body asymmetry in athletes with unilateral amputation, as well as muscle atrophy and altered extracellular fluid distribution in the lower limbs of athletes with spinal cord injury, factors that could reduce the accuracy of this technique in this athletic population.

As far as ADP is concerned, the results showed that %FM estimated by ADP was significantly lower than %FM_DXA, with poor agreement between measurements in both male and female PA (Table 3, Figure 3 and Figure 4). These results expand on previous findings in male wheelchair game players [32] and suggest that ADP is not a valid method for estimating body composition in Paralympians, with its outcomes requiring cautious interpretation.

The Bland–Altman analyses also revealed that the 95% limits of agreement were consistently wide across methods and subgroups. Numerically, the limits ranged from 7.47 to 26.34%FM in male PA and from 10.51 to 25.90%FM in female PA, with similarly broad confidence intervals (Table 3). These values indicate that an individual athlete’s %FM estimate obtained through field methods may deviate from their DXA-derived value by approximately ± 4–13%FM for the equations with narrower limits, and by ± 12–22%FM for those with poorer agreement (e.g., THO3, THO7, BIA and ADP). From a clinical and monitoring perspective, such variability is unlikely to be acceptable. In Paralympic athletes, meaningful within-athlete changes in adiposity are generally small (typically in the order of 1–3%FM), meaning that limits of agreement exceeding ± 7–10%FM markedly surpass the magnitude of changes that would be considered physiologically relevant or actionable. These findings suggest that none of the tested field methods provide agreement with DXA that is sufficiently narrow for individual-level monitoring. Although they may offer approximate group-level estimates, their ability to detect small but meaningful changes over time remains limited in this population.

In light of these findings, it is important to translate the observed methodological limitations into practical guidance for practitioners working with Paralympic athletes. Although DXA remains the preferred option whenever precise absolute estimates are required, such as at pre-season baseline or after targeted interventions, field methods still play a role in routine, higher-frequency monitoring. The typical error expectations observed in our sample indicate that skinfold-based %FM estimates generally show limits of agreement of approximately ±7–12%FM, BIA exhibits substantially wider variability (often ±12–22%FM), and ADP tends to provide more consistent yet still relatively wide limits (±6–10%FM). These magnitudes confirm that absolute %FM values derived from field techniques should be interpreted with caution. However, summed skinfolds may still be useful for detecting within-athlete trends over time, particularly when changes are gradual but meaningful for performance or health.

When it is not feasible to collect all nine skinfold sites, because of time constraints or impairment-related limitations such as altered positioning, asymmetry, or inaccessible anatomical landmarks, a reduced set can offer a pragmatic alternative. In these situations, triceps, subscapular, and thigh sites represent a reasonable compromise, although in athletes with high-level spinal cord injury the lower-limb sites may be impractical and upper-body sites become a more viable option. Likewise, in athletes with lower-limb amputations, thigh and calf measurements may be unreliable, making upper-body sites more informative. Collectively, these considerations highlight that while absolute %FM estimation using field methods is imprecise, carefully selected skinfold assessments, and particularly summed measures, remain valuable for longitudinal monitoring when DXA is unavailable.

The interplay of multiple factors influencing body composition in this unique athletic population, such as sex, race, underlying health condition (e.g., spinal cord injury, limb/s amputation/s), level and asymmetry of lesion, practiced sport and level of participation, makes the development of population-specific equations particularly challenging. In this study, we aimed to advance the development of specific equations for PA by analyzing male and female athletes separately and by providing data from a sample homogeneous in race and athletic level (all athletes being Paralympians). The correlation analysis showed that in male PA, the correlation coefficients between %FM_DXA and the biceps, chest, subscapular, anterior suprailiac, and abdominal skinfolds were near or slightly above 0.8 (Table 4 and Figure 5). In female PA, the anterior suprailiac, axillary, abdominal, and calf skinfolds significantly correlated with %FM_DXA, with correlation coefficients near or slightly above 0.8 (Table 4 and Figure 5). These results may reflect sex-specific patterns of fat distribution. In this context it is also interesting to underlay that in both male and female athletes, the sum of nine skinfolds is able to predict more than 80% of variance in predicting the %FM_DXA. In male PA, the linear regression model which uses the sum of nine skinfolds as independent variable and %FM_DXA as dependent variable showed an adjusted R^2^ near 0.80, with a relatively small SEE (~1.5). 1. Theoretically, as already reported by Goosey-Tolfrey et al. [32], an equation that includes a broader range of sites could better account for inter-individual variation in fat distribution. However, it should be noted that this potential advantage may be constrained by practical limitations when assessing certain lower-body sites in PA. In practical settings, the feasibility of collecting all nine skinfold sites may be limited in some impairment types due to restricted positioning, altered morphology, or inaccessibility of specific anatomical landmarks. For this reason, we also examined reduced-site alternatives (seven-, four-, and three-site models). Although these equations showed slightly higher SEE values compared to the nine-site model, their accuracy remained acceptable for applied use, particularly when clinicians or support staff must work under time constraints or adapt measurements to athletes’ functional limitations. In several cases, such as individuals with high-level spinal cord injury or limb deficiencies, reduced-site protocols may represent a realistic and pragmatic compromise, allowing routine body composition monitoring even when the standard nine-site assessment is not feasible. The cross-validation results confirm that the summed skinfold measures (three, seven, and nine sites) and the biceps skinfold are the most reliable predictors of %FM in both male and female athletes. Single skinfolds, particularly thigh and calf, and circumference measures showed lower predictive performance, highlighting that relying on isolated measurements may reduce accuracy. Notably, some differences between males and females were observed, suggesting that predictive performance may vary by sex. Overall, these findings support the use of summed skinfolds as preferred predictors for estimating %FM in Paralympic athletes. Taken together, these results may be useful in clinical settings, as they can help the guide of nutritionists, sports physicians, and physical trainers in selecting anatomical sites and determining the number of skinfolds to include in the assessments, providing valuable information on the nutritional status of athletes of both genders when DXA is unavailable.

Although the relatively large number of PA enrolled in this study, we were unable to perform statistical analyses by underlying health condition within each male and female group due to the relatively small sample sizes in each subgroup. However, we made an effort to provide informative results stratified by both sex and health condition (Table 1 and Figure 2, Figure 3 and Figure 4), offering guidance for professionals in assessing body composition in PA, given the lack of other published information. In fact, despite the importance of accurately assessing body composition in this special athletic population, the accuracy of the currently available methods for estimating body composition in PA relative to DXA has been poorly investigated to date [19,32,34,35,36]. These studies assessed the accuracy of anthropometry [19,32,34,35,36], BIA [32,34], and ADP [32] in relatively small and often heterogeneous samples of PA. For example, one study included 8 male and 8 female highly active athletes with spinal cord injury from a university team [34]; another involved 19 female wheelchair game players, comprising both athletes who were ambulant with support and those who were non-ambulant during daily living [19]; a third study examined 14 male elite wheelchair game players, either ambulant with support or non-ambulant during daily living [36]; finally, a further study evaluated 30 male trained wheelchair game players alongside 29 male athletes with unilateral lower limb amputation practicing a variety of adapted sports [35]. Based on the above, and to the best of our knowledge, the present study represents a first attempt to address gaps in the literature, where previous research often involved small numbers of athletes, and/or evaluated the accuracy of only a single field method for assessing body composition versus DXA (e.g., skinfold equations only).

This study has some limitations that should be acknowledged. First, as noted above, the relatively small number of athletes within each health condition group prevented us from considering the combined effect of gender and health condition on the accuracy of field methods for estimating %FM_DXA. Moreover, although descriptive results were stratified by health condition group, the sample size did not allow the estimation of agreement statistics with sufficient precision within each group. Future studies with larger sample sizes are therefore required to determine whether the performance of field methods varies across health condition groups. Second, the results obtained do not take into account the type of sport practiced by the athletes, which could influence the pattern of fat mass distribution. We aim in the future to increase the size of the study group in order to take into account all the different conditions, for example, the sport practiced, and their influence on body composition. Third, including only White participants in the analysis limits the applicability of the findings to the broader international Paralympic community. Therefore, future research on this topic is needed to also consider the impact of race on methodological issues related to body composition in Paralympic athletes worldwide. Fourth, residual edema, autonomic dysregulation, or extracellular fluid shifts, particularly in athletes with SCI, may influence BIA- or ADP-based estimates of body composition. Although standardized pre-test procedures were applied, including fasting and bladder voiding, these physiological factors could introduce variability in the measurements and should be considered when interpreting the results. Further research with a larger sample size would allow these athletes to be considered separately and provide more specific insights into the impact of these factors. Furthermore, although impairment groups were descriptively reported, the sample size did not permit meaningful sensitivity or stratified analyses that could clarify whether heterogeneity related to impairment type, ambulatory status, or sport classification influences the performance of the field methods. This heterogeneity is intrinsic to Paralympic populations and may affect both the distribution of fat mass and the assumptions underlying predictive models. Future studies with larger and more balanced samples are therefore required to formally evaluate the impact of these factors on methodological accuracy.

However, a strength point of this study was that we considered three currently used methods that can be available to assess body composition in this athletic population when DXA cannot be used (i.e., anthropometry, BIA and ADP). A further strength of this study is that we provided data on a relatively large sample of male and female PA, all at the elite level.

In conclusion, the results of this study indicate a lack of transferability of available methods for assessing body composition (skinfold equations, BIA, and ADP) in estimating %FM compared to DXA in both male and female Paralympians, as these methods proved inaccurate. Given the importance of providing clinicians with accessible field-based methods to assess body composition in this special athletic population, future research should focus on developing approaches that integrate different factors, such as gender and type of impairment. The sum of skinfolds may represent a suitable option, particularly when combined with body circumferences. Although existing skinfold equations show limited accuracy compared to DXA, with large differences observed within para-athletes, and results should therefore be interpreted with caution, the sum of skinfolds can still be useful for monitoring individual changes in body composition throughout the season.

## Figures and Tables

**Table 1:** Skinfold equations used in this study to predict body density or relative fat mass in Paralympians.

References	Abbreviation	Sex	Skinfold Equation
Durnin and Womersley, 1974 [40]	%FM_DW	M	BD = 1.1765 − [0.0744 · log10 (BI + TR + SS + SI)]
		F	BD = 1.1567 − [0.0717 · log10 (BI + TR + SS + SI)]
Evans et al., 2005 [39]	%FM_EV3	M/F	%FM = 8.997 + [0.24658 · (TR + AB + TH)] − (6.343 · Sex) − (1.998 · Race)
Evans et al., 2005 [39]	%FM_EV7	M/F	%FM = 10.566 + [0.12077 · (TR + SI + TH + SS + AX + CH + AB)] − (8.057 · Sex) − (2.545 · Race)
Pollock et al., 1976 [41]	%FM_POL	M	BD = 1.09716 − (0.00065 · CH) − (0.00055 · SS) − (0.0008 ·TH)
		F	BD = 1.0852 − (0.0008 · SI) − (0.00011 · TH)
Thorland et al., 1984 [42]	%FM_THO3	M	BD = 1.1136 − [0.00154 · (TR + SS + AX)] + [0.00000516 · (TR + SS + AX)^2^]
		F	BD = 1.0987 − [0.00122 · (TR + SS + SI)] + [0.00000263 · (TR + SS + SI)^2^]
Thorland et al., 1984 [42]	%FM_THO7	M	BD = 1.1091 − [0.00052 · (TR + SS + AX + SI + AB + TH + CA)] + [0.00000032 · (TR + SS + AX + SI + AB + TH + CA)^2^]
		F	BD = 1.1046 − [0.00059 · (TR + SS + AX + SI + AB + TH + CA)] + [0.00000060 · (TR + SS + AX + SI + AB + TH + CA)^2^]

Legend: BD, body density; %FM, relative fat mass; BI, biceps; TR, triceps; SS, subscapular; SI, suprailiac; TH, thigh; AB, abdominal; AX, axilla; CA, calf; CH, chest; Sex, male = 1 and female = 0; Race, black = 1 and white = 0.

**Table 2:** General characteristics of athletes stratified by gender and health conditions. Data are presented as Mean and standard deviation (SD).

		Males		Females	
Variable	Group	Mean	SD	Mean	SD
Age (years)	SCI	35.3	10.7	35.0	10.1
	AMP	29.1	6.4	29.5	8.0
	BI	32.3	9.3	27.3	2.1
	OTH	32.6	9.3	35.5	10.2
	VI	24.0	4.4	31.1	10.5
Body Mass (kg)	SCI	69.1	11.5	58.2	8.0
	AMP	68.1	14.6	56.5	7.7
	BI	65.9	8.5	67.4	17.7
	OTH	68.4	16.1	67.4	10.7
	VI	83.3	11.8	74.1	26.8
Stature (cm)	SCI	175.0	12.9	166.3	9.9
	AMP	173.1	17.5	166.3	4.9
	BI	176.4	8.4	166.3	8.4
	OTH	176.6	7.0	168.1	7.8
	VI	178.8	1.9	171.2	8.3
BMI (kg/m^2^)	SCI	22.8	5.0	21.1	3.1
	AMP	22.8	3.8	20.4	2.2
	BI	21.1	1.8	24.1	3.7
	OTH	21.8	4.3	23.9	4.0
	VI	26.1	4.2	24.8	6.7

Legend: SCI, Spinal Cord Injuries and Related Disorders Group; AMP, Amputation Group; BI, Brain Injury Group; OTH, Other Health Conditions Group; VI, Vision and Intellectual Health Conditions Group; BMI, Body Mass Index.

**Table 4:** Linear regression parameters and 5-fold cross-validation performance for all anthropometric and body composition predictors of %FM (DXA as criterion measure).

	Males									Females								
	Linear Regression Analysis							Cross-Validation		Linear Regression Analysis							Cross-Validation	
Variable	*n*	r_P_/r_s_	Adj R^2^	SEE	Int	Coe	*p*	RMSPE (M ± SD)	R^2^ (CV) (M ± SD)	*n*	r_P_/r_s_	Adj R^2^	SEE	Int	Coe	*p*	RMSPE (M ± SD)	R^2^ (CV) (M ± SD)
Biceps SKF	62	0.86	0.74	3.39	11.70	2.39	<0.001	3.40 ± 0.69	0.76 ± 0.03	64	0.68	0.43	4.54	22.74	1.15	<0.001	4.65 ± 0.48	0.51 ± 0.14
Triceps SKF	63	0.72	0.50	4.66	14.57	0.87	<0.001	4.54 ± 1.34	0.55 ± 0.18	63	0.65	0.40	4.63	21.53	0.54	<0.001	4.75 ± 1.34	0.55 ± 0.19
Chest SKF	34	0.75	0.55	2.47	13.87	1.17	<0.001	2.64 ± 0.50	0.53 ± 0.30	43	0.73	0.50	3.99	20.88	1.42	<0.001	3.89 ± 0.66	0.53 ± 0.05
Subscapular SKF	62	0.75	0.56	4.39	13.98	0.69	<0.001	4.16 ± 1.74	0.56 ± 0.32	62	0.73	0.51	4.18	22.76	0.61	<0.001	4.19 ± 0.58	0.55 ± 0.14
Anterior suprailiac SKF	31	0.83	0.68	2.50	13.91	0.83	<0.001	2.44 ± 0.59	0.69 ± 0.22	44	0.81	0.55	3.71	23.10	0.70	<0.001	3.65 ± 0.34	0.55 ± 0.11
Mid-suprailiac SKF	60	0.74	0.53	4.27	14.16	0.61	<0.001	4.16 ± 1.52	0.59 ± 0.25	62	0.72	0.51	4.17	22.23	0.57	<0.001	4.19 ± 0.29	0.56 ± 0.11
Axillary SKF	30	0.59	0.33	3.19	14.54	0.75	0.001	3.62 ± 0.98	0.35 ± 0.31	39	0.84	0.71	2.89	19.86	1.00	<0.001	3.21 ± 0.92	0.80 ± 0.07
Abdominal SKF	30	0.79	0.61	2.41	13.92	0.51	<0.001	2.55 ± 0.46	0.53 ± 0.35	40	0.84	0.59	3.45	19.28	0.72	<0.001	3.60 ± 0.91	0.66 ± 0.16
Thigh SKF	28	0.67	0.42	3.04	15.07	0.43	<0.001	3.23 ± 1.39	0.61 ± 0.31	39	0.59	0.28	4.63	20.76	0.35	<0.001	4.70 ± 0.90	0.37 ± 0.19
Calf SKF	24	0.55	0.28	2.67	16.03	0.49	0.005	2.74 ± 0.68	0.55 ± 0.37	37	0.77	0.59	3.31	20.27	0.59	<0.001	3.10 ± 1.11	0.62 ± 0.21
Sum of three SKFs	62	0.85	0.71	3.58	11.41	0.41	<0.001	3.65 ± 0.92	0.70 ± 0.14	61	0.77	0.58	3.81	19.49	0.29	<0.001	3.76 ± 0.63	0.58 ± 0.20
Sum of four SKFs	60	0.79	0.73	3.25	11.56	0.26	<0.001	3.33 ± 0.71	0.71 ± 0.23	61	0.82	0.62	3.64	19.19	0.21	<0.001	3.58 ± 0.81	0.66 ± 0.12
Sum of seven SKFs	29	0.74	0.69	2.20	10.79	0.15	<0.001	2.21 ± 0.54	0.71 ± 0.12	39	0.89	0.77	2.56	16.81	0.15	<0.001	2.49 ± 0.53	0.81 ± 0.05
Sum of nine SKFs	24	0.88	0.76	1.54	10.92	0.11	<0.001	1.60 ± 0.32	0.84 ± 0.08	37	0.90	0.79	2.37	15.18	0.11	<0.001	2.39 ± 0.45	0.84 ± 0.01
Waist circumference	30	0.48	0.20	3.47	2.96	0.22	0.008	3.36 ± 0.83	0.25 ± 0.20	41	0.75	0.49	3.95	6.33	0.32	<0.001	4.05 ± 0.68	0.60 ± 0.10
Abdominal circumference	30	0.46	0.18	3.50	2.32	0.22	0.011	3.63 ± 0.91	0.24 ± 0.24	40	0.48	0.21	4.93	18.78	0.15	0.002	5.51 ± 2.60	0.56 ± 0.31
Hip circumference	21	0.24	0.01	3.18	11.01	0.91	0.292	3.22 ± 0.85	0.29 ± 0.33	33	0.72	0.50	3.76	−13.95	0.45	<0.001	3.69 ± 1.04	0.59 ± 0.25

Legend: SKF, skinfold; r_P_, Pearson product–moment correlation coefficient; r_s_, Spearman rank correlation coefficient; SEE, standard error of estimate; Int, Intercept; Coe, Coefficient (β); *p*, *p*-value; RMSPE, root mean square prediction error; R^2^ (CV), coefficient of determination obtained from 5-fold cross-validation; M, mean; SD, standard deviation; Sum of three SKFs, sum of biceps, triceps, subscapular skinfolds; Sum of four SKFs, sum of biceps, triceps, subscapular, and mid-suprailiac skinfolds; Sum of seven SKFs, sum of biceps, triceps, subscapular, mid-suprailiac, chest, axillary, and abdominal skinfolds; Sum of nine SKFs, sum of biceps, triceps, subscapular, mid-suprailiac, chest, axillary, abdominal, thigh, and calf skinfolds.

## Data Availability

The raw data supporting the conclusions of this article will be made available by the authors on request.

## Abbreviations

The following abbreviations are used in this manuscript:

PA	Paralympic athletes
DXA	Dual-Energy X-ray Absorptiometry
BIA	Bioelectrical Impedance Analysis
ADP	Air Displacement Plethysmography
%FM_DXA	DXA-assessed whole-body relative fat mass
SKFs	skinfolds
%FM_BIA	BIA-assessed whole-body relative fat mass
%FM_ADP	ADP-assessed whole-body relative fat mass
%FM_DW	%FM estimated by the Durnin and Womersley equation
%FM_EV3	%FM estimated using the three-site equation by Evans and colleagues
%FM_EV7	%FM estimated using the seven-site equation by Evans and colleagues
%FM_POL	%FM estimated using the equation by Pollock and colleagues
%FM_THO3	%FM estimated using the three-site equation by Thorland and colleagues
%FM_THO7	%FM estimated using the seven-site equation by Thorland and colleagues
SCI	Spinal Cord Injuries and Related Disorders Group
AMP	Amputation Group
BI	Brain Injury Group
OTH	Other Health Conditions Group
VI	Vision and Intellectual Health Conditions Group
BMI	Body Mass Index
r_P_	Pearson product–moment correlation coefficient
r_s_	Spearman rank correlation coefficient
*p*	*p*-value
